# Associations between the patient-centered medical home and preventive care and healthcare quality for non-elderly adults with mental illness: A surveillance study analysis

**DOI:** 10.1186/s12913-016-1676-z

**Published:** 2016-08-24

**Authors:** Jennifer J. Bowdoin, Rosa Rodriguez-Monguio, Elaine Puleo, David Keller, Joan Roche

**Affiliations:** 1 Health Policy and Management, School of Public Health and Health Sciences, University of Massachusetts Amherst, 715 North Pleasant Street, Amherst, MA 01003 USA; 2 Department of Pediatrics, University of Colorado School of Medicine, 13123 E. 16th Avenue, B065, Aurora, CO 80045 USA; 3 The Roche Associates, 37 Westmoreland Avenue, Longmeadow, MA 01106 USA; 4 School of Public Health and Health Sciences, University of Massachusetts Amherst, 715 North Pleasant Street, Amherst, MA 01003 USA

**Keywords:** Patient-centered medical home, Mental illness, Healthcare quality, Preventive care, Medical expenditure panel survey

## Abstract

**Background:**

Patient-centered medical homes (PCMHs) may improve outcomes for non-elderly adults with mental illness, but the extent to which PCMHs are associated with preventive care and healthcare quality for this population is largely unknown. Our study addresses this gap by assessing the associations between receipt of care consistent with the PCMH and preventive care and healthcare quality for non-elderly adults with mental illness.

**Methods:**

This surveillance study used self-reported data for 6,908 non-elderly adults with mental illness participating in the 2007–2012 Medical Expenditure Panel Survey. Preventive care and healthcare quality measures included: participant rating of all healthcare; cervical, breast, and colorectal cancer screening; current smoking; smoking cessation advice; flu shot; foot exam and eye exam for people with diabetes; and follow-up after emergency room visit for mental illness. Multiple logistic regression models were developed to compare the odds of meeting preventive care and healthcare quality measures for participants without a usual source of care, participants with a non-PCMH usual source of care, and participants who received care consistent with the PCMH.

**Results:**

Compared to participants without a usual source of care, those with a non-PCMH usual source of care had better odds of meeting almost all measures examined, while those who received care consistent with the PCMH had better odds of meeting most measures. Participants who received care consistent with the PCMH had better odds of meeting only one measure compared to participants with a non-PCMH usual source of care.

**Conclusions:**

Compared with having a non-PCMH usual source of care, receipt of care consistent with the PCMH does not appear to be associated with most preventive care or healthcare quality measures. These findings raise concerns about the potential value of the PCMH for non-elderly adults with mental illness and suggest that alternative models of primary care are needed to improve outcomes and address disparities for this population.

**Electronic supplementary material:**

The online version of this article (doi:10.1186/s12913-016-1676-z) contains supplementary material, which is available to authorized users.

## Background

Approximately 44 million (18.5 %) adults in the United States (US) have a mental illness [1]. Adults with mental illness have poorer health and social outcomes than adults without mental illness [2–4]. The patient-centered medical home (PCMH) has gained substantial attention as a promising strategy to address many shortcomings of the US healthcare system [5–8], including those that contribute to the poor outcomes of people with mental illness [9, 10]. Originally developed in the 1960s as a model for improving coordination of care for children with complex needs [11], the PCMH has evolved into an approach to primary care that is comprehensive, patient-centered, coordinated, accessible, and committed to quality and safety [12]. When fully implemented, the PCMH is expected to achieve the Institute for Healthcare Improvement’s Triple Aim of improved patient experience, improved health, and reduced costs [13].

Prior systematic reviews have found mixed results for the PCMH overall [14–18], but three retrospective cohort studies conducted with non-elderly adults with mental illness enrolled in the North Carolina Medicaid program indicated that the PCMH may have favorable effects on medication adherence [19, 20], preventive screenings [20], and outpatient follow-up after psychiatric discharge [21]. A study conducted with 9,303 North Carolina Medicaid-enrolled non-elderly adults with depression and at least one other chronic condition found that enrollees who had a PCMH had better rates of antidepressant adherence than those without a PCMH [19]. A second study conducted with 7,228 adults with schizophrenia, 13,406 adults with bipolar disorder, and 45,000 adults with major depression reported that North Carolina Medicaid enrollees with a PCMH had better rates of medication adherence than those who did not have a PCMH [20]. The authors also found that, among participants with major depression, having a PCMH was associated with better rates of lipid and cancer screening. A third study conducted with North Carolina Medicaid-enrolled non-elderly adults with multiple chronic conditions and a hospitalization for either schizophrenia (*N* = 8,783) or depression (*N* = 18,658) found that those with a PCMH were more likely to receive follow-up care with any provider and with a primary care provider within 30 days post-discharge [21]. To our knowledge, no other peer-reviewed studies have examined the association between the PCMH and receipt of recommended preventive care or better healthcare quality for non-elderly adults with mental illness. Thus, additional research in this area is needed. This study addresses this gap by examining the association between receipt of care consistent with the PCMH and preventive care and healthcare quality measures for a nationally representative sample of non-elderly adults with mental illness in the United States. In doing so, this study will help providers, policymakers, and payers assess whether the PCMH is an effective model for improving healthcare quality and outcomes for non-elderly adults with mental illness.

## Methods

### Hypotheses

We hypothesized that receipt of care consistent with the PCMH is positively associated with receipt of recommended preventive care and better healthcare quality for non-elderly adults with mental illness.

### Study design

We conducted a surveillance study analysis using secondary data from five Medical Expenditure Panel Survey (MEPS) cohorts. MEPS is a set of large-scale surveys that provide nationally representative estimates for socio-economic, demographic, health, and healthcare characteristics for the US civilian non-institutionalized population [22, 23]. MEPS uses a panel design with five rounds of interviews and supplemental surveys that cover two calendar years for each cohort.

### Data sources

Study data were collected through the MEPS Household and Medical Provider components. The Household Component includes detailed data at the individual and household levels on a broad range of health-related variables [23]. The Medical Provider Component supplements and/or replaces medical event information reported by respondents with information provided by a sample of participants’ providers. Study data were derived primarily from the Longitudinal Data Files for panels 12 (2007–2008) through 16 (2011–2012). The Longitudinal Data File for each panel is a 2 year file that contains data from rounds 1–5 for individuals who were in-scope (i.e., non-institutional civilian population) and had data collected in all MEPS rounds that they participated [23]. Data on clinical conditions were obtained from the 2007–2012 Medical Conditions Data Files. Data on medical care events were obtained from the 2007–2012 Hospital Inpatient Stays Files, the 2007–2012 Emergency Room Visits Files, the 2007–2012 Outpatient Visits Files, and the 2007–2012 Office-Based Medical Provider Visits Files.

### Participants

This study included MEPS participants in panels 12–16 who were 18–64 years old, were in-scope in all survey rounds, and had data collected in all survey rounds.

The overall MEPS survey response rate for the full sample eligible for participation in MEPS panels 12–16 ranged from 52 to 62 % [24].

### Mental illness status

Based on SAMHSA’s definition [1], mental illness was defined as a mental, behavioral, or emotional disorder, other than a developmental or substance use disorder. Study participants were classified as having mental illness if they had any of the following types of conditions in any survey round: adjustment disorders; anxiety disorders; delirium, dementia, and amnestic disorders; impulse control disorders; mood disorders; personality disorders; schizophrenia and other psychotic disorders; and miscellaneous mental disorders. Conditions identified in MEPS include those linked to an event or disability day or a condition the person was experiencing during the survey period [25]. Physical and behavioral health conditions were coded using International Classification of Diseases, Ninth Revision, Clinical Modification codes and subsequently aggregated into clinically meaningful categories.

### Provider type

Participants were first assessed to determine whether they received care consistent with the PCMH, had a non-PCMH usual source of care (USC), or did not have a USC in each study year. Participants were determined to have a USC if they reported that: 1) they had a particular place they usually went to when sick or needed advice about health; and 2) the place was a location other than an ER. Using AHRQ’s definition [12] as the basis, participants who had a USC were classified as receiving care consistent with the PCMH if they reported that the USC provided comprehensive, patient-centered, and accessible care (Table 1); two attributes in AHRQ’s definition, coordinated care and a commitment to quality and safety, cannot be assessed through MEPS and were not included in this study’s definition.

**Table 1:** PCMH Model and Attribute Definitions

PCMH Attribute	PCMH Characteristic^a^	Allowable Responses
Received comprehensive care	USC usually asked about medications and treatments prescribed by other doctors [26, 27, 29]	Yes
	USC provided care for new health problems [26–28]	Yes
	USC provided preventive healthcare [26–28]	Yes
	USC provided referrals to other health professionals [26–28]	Yes
	USC provided care for ongoing health problems [26, 27]	Yes
	*Participant received comprehensive care* [26]	*Yes to all five questions*
Received patient-centered care	USC showed respect for the medical, traditional, and alternative treatments with which participant is happy [26]	Usually, always
	USC asked participant to help decide treatment when there was a choice of treatments [26–29]	Usually, always
	USC presented and explained all healthcare options to participant [26]	Yes
	*Participant received patient*-*centered care* [26]	*Usually*, *always*, *or yes to all three questions*
Received accessible care	It was not difficult to get to USC’s location	Not too difficult, not at all difficult
	It was not difficult to contact USC over the phone about a health problem during regular office hours [26, 27, 29]	Not too difficult, not at all difficult
	USC offered night and weekend office hours [26–29]	Yes
	USC spoke the participant’s preferred language or provided translation services [26]	Yes
	*Participant received accessible care*	*Not too difficult*, *not at all difficult*, *or yes to all four questions*
Received care consistent with the PCMH	*Participant had a USC other than an ER that provided comprehensive*, *patient*-*centered*, *and accessible care* [26, 28, 29]	*Allowable responses for all attributes*

^a^ Prior research that informed the selection of MEPS variables is cited as appropriate, but there were some coding and other differences between this study and how the cited studies assessed PCMH characteristics. Additional methodological details are available from the authors upon request

MEPS variables used to assess whether participants received care consistent with the PCMH were selected based on face validity, prior MEPS research [26–29], and feasibility for use in this study. Comprehensive care was determined based on whether: 1) the USC usually asked about medications and treatments prescribed by other doctors; and the USC provided: 2) care for new health problems; 3) preventive healthcare; 4) referrals to other health professionals when needed; and 5) care for ongoing health problems. A USC that met all five criteria was deemed comprehensive. Patient-centered care was assessed based on whether the provider: 1) usually or always showed respect for the medical, traditional, and alternative treatments with which the participant was happy; 2) usually or always asked the participant to help decide the treatment when there was a choice; and 3) presented and explained all healthcare options to the participant. A USC that met all three criteria was coded patient-centered. Accessible care was evaluated based on whether the USC: 1) was not too difficult or not at all difficult to get to; 2) was not too difficult or not at all difficult to contact via phone during regular office hours; 3) offered night and weekend office hours; and 4) spoke the participant’s preferred language or provided translation services. A USC that met all four criteria was coded accessible.

Participants with responses of don’t know, refused, or not ascertained to the question about whether they had a USC (*N* = 118, 0 %) were excluded from the final analytical sample. Participants were also excluded if a response was refused or not ascertained (*N* = 27, 0 %) or if a proxy respondent did not know the answer (*N* = 742, 1 %) to any PCMH question except the question about preferred language, as MEPS does not collect this information on participants who are comfortable conversing in English; participants who had a USC and were comfortable conversing in English were coded as having a USC who spoke the participant’s preferred language. One percent of participants (*N* = 883) were excluded from the final analytical sample because of missing PCMH data.

Participants who did not have a proxy respondent and did not know the answer to any of the comprehensive, patient-centered, and accessible care questions (*N* = 1,682; 26 % of the final analytical sample) were coded as not receiving the characteristic; 1,025 participants (17 % of the final analytical sample) had one or more variables recoded in year 1 and 1,043 participants (16 % of the final analytical sample) had one or more variables recoded in year 2 for this reason. For most variables, 0-4 % of the final analytical sample was recoded because a proxy respondent did not know whether the USC met the characteristic. The only variables with a sizable number of participants recoded were those that examined how often the provider showed respect for the medical, traditional, and alternative treatments with which the participant was happy (year 1: *N* = 449, 7 %; year 2: *N* = 457, 7 %) and whether the USC offered night and weekend office hours (year 1: *N* = 363, 6 %; year 2: *N* = 406, 6 %).

Once participants were classified by provider type in each study year, this information was used to determine whether participants received care consistent with the PCMH, had a non-PCMH USC, or did not have a USC in at least 1 year and in both years. Participants were first assessed to determine if they received care consistent with the PCMH in at least 1 year and if they received care consistent with the PCMH in both years. Participants who did not receive care consistent with the PCMH in either year but reported having a USC in at least 1 year were classified as having a non-PCMH USC in at least 1 year. Participants who did not receive care consistent with the PCMH in both years but reported having a USC in both years were classified as having a non-PCMH USC in both years; some participants classified as having a non-PCMH USC in both years received care consistent with the PCMH in one but not both years (*N* = 981; 20 % of participants classified as having a non-PCMH USC in both years). Participants who did not report having a USC in either year were classified as not having a USC in at least 1 year. Participants who did not have a USC in one or both years were classified as not having a USC in both years; some participants classified as not having a USC in both years received care consistent with the PCMH (*N* = 163; 9 % of participants classified as not having a USC in both years) or had a USC (977; 53 % of participants classified as not having a USC in both years) in one but not both years.

### Preventive care and healthcare quality measures

Preventive care and healthcare quality measures included a healthcare rating measure, as recommended by the Institute for Healthcare Improvement (n.d.a) and prevention and condition-specific measures adapted National Quality Forum-endorsed measures (Table 2). The measures were comprised of a participant rating of all healthcare, three cancer screening measures (cervical, breast, colorectal), a measure to assess current smoking, a smoking cessation advice measure, a flu shot measure, two diabetes-specific measures (foot exam, eye exam), and follow-up after an emergency room (ER) visit for mental illness. Participants were assessed in each year to determine if they met the measure, using the relevant look-back period identified in Table 2. For instance, the measure description for cervical cancer screening is “Had most recent pap test within past 3 years.” In each study year, women who met the inclusion criteria for this measure (i.e., age 23–62, had not had a hysterectomy at any time) were assessed to determine whether they reported having a pap test within the past 3 years.

**Table 2:** Preventive Care and Healthcare Quality Measures

Preventive Care and Quality Measure	Measure Description	Eligible Participants	Sample Sizes Included in Analyses
Healthcare rating	Gave all healthcare in last 12 months a 9 or 10 rating on a scale of 0 (worst) to 10 (best)	Participants who received healthcare in last 12 months	4773
Cervical cancer screening	Had most recent pap test within past 3 years	Women age 23–62 who have not had a hysterectomy at any time	3133
Breast cancer screening	Had most recent mammogram within past 2 years	Women age 51 and older	1461
Colorectal cancer screening	Had most recent blood stool test within past year, most recent colonoscopy within past 10 years, or most recent sigmoidoscopy within past 5 years	Participants age 50 and older in panels 14–16 who have not had colon or rectal cancer at any time^a^	1531
Current smoking	Smoked at the time the survey was conducted	All participants	6697
Smoking cessation advice	Doctor advised to quit smoking in last 12 months^b^	Current smokers	1382
Flu shot	Had flu shot within past year	All participants	6758
Foot exam	Health professional checked feet for sores or irritations at least once in past year	Participants with diabetes	640
Eye exam	Had dilated eye exam within past year	Participants with diabetes	643
Follow-up after emergency room (ER) visit for mental illness	Had at least one office-based provider or hospital outpatient visit with any provider with a primary diagnosis of mental illness within 7 days of the first ER visit with a primary diagnosis of mental illness	Participants with an ER visit^c^ with a primary diagnosis of mental illness	245

^a^ Data is not available in MEPS to measure receipt of colorectal cancer screening, as defined in this study, for panels 12 and 13

^b^ Participants who did not have any visits to a doctor in the last 12 months were coded as not receiving smoking cessation advice

^c^ Excludes ER visits that occurred after the first 11 months of the second measurement year, as well as ER visits followed by an ER visit for mental health or a hospitalization for any reason within the follow-up period

All preventive care and quality measures were constructed as dichotomous variables that separately examined whether the participant met the criteria for the measure in at least 1 year and in both years. The healthcare rating measure asked respondents to rank all of their healthcare on a scale of 0–10, with 0 being the worst healthcare possible and 10 being the best healthcare possible; participants with a rating of 9 or 10 on this measure were identified as receiving good quality healthcare [30]. Participants with responses of don’t know, refused, or not ascertained in one or both survey years were excluded from the condition-specific analyses. For most measures, 0-5 % of eligible participants were excluded; 7 % (*N* = 199) of eligible participants were excluded from the smoking cessation advice analyses because required data was not obtained through the Self-Administered Questionnaire, a supplemental paper-based survey.

### Covariates

Multivariate logistic regression models included the following covariates: age, gender, race/ethnicity, immigration status, language, marital status, lived alone, other smokers in the household (current smoking measure only), education, family income, employment, received Supplemental Security Income due to disability, geographic location, urban residence, health insurance, disability days, substance use disorder diagnosis, medical comorbidity score [31, 32], activity of daily living limitation, instrumental activity of daily living limitation, psychological distress [33], mental health status [34], physical health status [34], MEPS survey panel, household interview proxy respondent, Self-Administered Questionnaire proxy respondent, Diabetes Care Survey proxy respondent (foot exam and eye exam measures only), anxiety disorder, mood disorder, schizophrenia and other psychotic conditions, and other mental disorder. The Self-Administered Questionnaire includes measures of psychological distress, mental health status, and physical health status [35]; MEPS participants who had missing data on these measures were excluded from the final analytical sample (*N* = 676; 1 %). Missing data for other covariates were classified unknown and included in the analyses; 486 participants (6 % of the final analytical sample) had missing data on one or more covariates classified as unknown.

### Analyses

Bivariate analyses were conducted to compare the characteristics of participants by provider type. Univariate analyses were conducted to examine the number and percentage of participants who had a USC, received each PCMH attribute, and received care consistent with the PCMH in at least 1 year and in both years. Bivariate analyses were used to assess the number and percentage of participants who met each preventive care and healthcare quality measure in at least 1 year and in both years, by provider type. Simple and multiple logistic regression models were developed to assess the odds of meeting each preventive care and healthcare quality measure, comparing participants with each provider type in at least 1 year and in both years individually with each other (e.g., participants with a non-PCMH USC in at least 1 year compared to participants without a USC in at least 1 year). Sensitivity analyses were also conducted, excluding participants who did not have the same provider type in both years (*N* = 2121; 30 %) from the multivariate analyses comparing participants with each provider type in both years.

Due to small sample sizes, some regression analyses were not valid. As a result, additional multiple logistic regression models were developed to assess the odds of meeting some preventive care and healthcare quality measures for participants who received care consistent with the PCMH compared to participants who did not receive care consistent with the PCMH. In these analyses, participants who did not receive care consistent with the PCMH included participants without a USC and participants with a non-PCMH USC. Similar to the main multiple regression models, these analyses were conducted comparing participants with each provider type in at least 1 year and/or in both years individually with each other (e.g., participants who received care consistent with the PCMH in at least 1 year compared to participants who did not receive care consistent with the PCMH in at least 1 year). However, these additional analyses focused on measures and time periods that could not be assessed in one or more of the main multivariate analyses because of small sample sizes (e.g., cervical cancer screening in at least 1 year but not in both years, smoking cessation advice in at least 1 year and in both years).

Significance levels were adjusted in multivariate analyses to account for multiple comparisons [36]. Longitudinal weights, which adjust for nonresponse and attrition when pooling multiple MEPS panels, and variance estimation variables, which account for complexity in MEPS sample design [23], were included in all analyses. Analyses were performed using Stata SE 13.1 [37]. All percentages displayed are weighted percentages.

## Results

### Study sample and characteristics

MEPS panels 12–16 included 80,001 people who were in-scope and had data collected in all rounds that they participated in the survey. Of them, 73,093 (90 %) were excluded: 24,796 (27 %) were under age 18; 8,479 (12 %) were over age 64; 38,092 (49 %) did not have mental illness; 307 (0 %) were not in-scope in all survey rounds; and 1,419 (2 %) were missing required data. The final study sample was comprised of 6,908 non-elderly adults with mental illness.

At least three-quarters of participants had a USC regardless of duration examined (*N* = 6,175, 90 % at least 1 year; *N* = 5,035, 75 % both years). Among those with a USC in at least 1 year, 88 % received comprehensive care, 81 % received patient-centered care, 35 % received accessible care, and 23 % received care consistent with the PCMH in at least 1 year (Table 3). Among those with a USC in both years, 63 % received comprehensive care, 53 % received patient-centered care, 14 % received accessible care, and 6 % received care consistent with the PCMH in both years. In total, 733 (10 %) participants did not have a USC, 4,709 (69 %) had a non-PCMH USC, and 1,466 (21 %) received care consistent with the PCMH in at least 1 year; 1,873 (25 %) did not have a USC, 4,713 (70 %) had a non-PCMH USC, and 322 (6 %) received care consistent with the PCMH in both years.

**Table 3:** Receipt of Care Consistent with the PCMH for Participants with a USC

PCMH Attribute	PCMH Characteristic	At Least One Year (*N* = 6175)	Both Years (*N* = 5035)
		N (weighted %)	
Comprehensive care	USC usually asked about medications and treatments prescribed by other doctors	5640 (91.7 %)	3486 (69.3 %)
	USC provided care for new health problems	6121 (99.1 %)	4832 (96.1 %)
	USC provided preventive healthcare	6094 (98.6 %)	4819 (95.6 %)
	USC provided referrals to other health professionals	6091 (98.5 %)	4773 (94.6 %)
	USC provided care for ongoing health problems	6093 (98.4 %)	4810 (95.1 %)
	*Participant received comprehensive care*	*5461* (*88.4* %)	*3186* (*62.9* %)
Patient-centered care	USC showed respect for the medical, traditional, and alternative treatments with which participant is happy	5619 (90.9 %)	3567 (70.7 %)
	USC asked participant to help decide treatment when there was a choice of treatments	5470 (89.5 %)	3391 (68.3 %)
	USC presented and explained all healthcare options to participant	5942 (96.1 %)	4329 (86.0 %)
	*Participant received patient*-*centered care*	*4989* (*81.0* %)	*2655* (*52.7* %)
Accessible care	It was not difficult to get to USC’s location	5914 (96.2 %)	4334 (87.2 %)
	It was not difficult to contact USC over the telephone about a health problem during regular office hours	5434 (88.4 %)	3350 (67.1 %)
	USC offered night and weekend office hours	2702 (43.5 %)	1052 (21.0 %)
	USC spoke the participant’s preferred language or provided translation services	6165 (99.9 %)	5012 (99.8 %)
	*Participant received accessible care*	*2181* (*35.3* %)	*683* (*13.8* %)
Received care consistent with the PCMH	*Participant had a USC other than an ER that provided comprehensive*, *patient*-*centered*, *and accessible care*	*1466* (*23.4* %)	*322* (*6.2* %)

With the exception of most comparisons examining differences in the prevalence of specific types of mental health and substance use disorder conditions, provider type was associated with all socio-demographic and health characteristics examined in the analyses comparing participants who did not have a USC to either those who had a non-PCMH USC or those who received care consistent with the PCMH (Table 4). There were significant differences in the prevalence of mood disorders between participants without a USC and those with a non-PCMH USC; no other comparisons in the prevalence of specific types of mental health conditions showed statistically significant differences between provider types. In contrast, only half of the comparisons between participants who had a non-PCMH USC and those who received care consistent with the PCMH were statistically significant. Specifically, comparisons between participants who had a non-PCMH USC and those who received care consistent with the PCMH were statistically significant for the following characteristics: gender (comparisons between provider types in at least 1 year only); marital status; education (comparisons between provider types in at least 1 year only); year 1 family income; year 2 family income (comparisons between provider types in at least 1 year only); employment; and year 1 and year 2 health insurance status.

**Table 4:** Characteristics of Study Participants by Provider Type

Characteristic	Provider Type N (weighted % or mean)				*P*-Value		
	Duration	No USC (At least 1 year: *N* = 733; Both years: *N* = 1873)	Non-PCMH USC (At least 1 year: *N* = 4709; Both years: *N* = 4713)	PCMH (At least 1 year: *N* = 1466; Both years: *N* = 322)	No USC vs. Non-PCMH USC	No USC vs. PCMH	Non-PCMH USC vs. PCMH
Age							
18-34 years	At least 1 year	400 (54.7 %)	1267 (27.3 %)	397 (27.3 %)	0.000	0.000	0.356
35-49 years		225 (29.3 %)	1661 (34.2 %)	544 (36.5 %)			
50-64 years		108 (16.0 %)	1781 (38.6 %)	525 (36.2 %)			
18-34 years	Both years	895 (46.8 %)	1087 (24.1 %)	82 (26.0 %)	0.000	0.000	0.412
35-49 years		616 (32.0 %)	1693 (34.8 %)	121 (37.1 %)			
50-64 years		362 (21.2 %)	1933 (41.1 %)	119 (36.8 %)			
Gender							
Male	At least 1 year	327 (48.1 %)	1405 (32.4 %)	405 (29.1 %)	0.000	0.000	0.032
Female		406 (51.9 %)	3304 (67.6 %)	1061 (70.9 %)			
Male	Both years	710 (41.4 %)	1346 (30.8 %)	81 (25.5 %)	0.000	0.000	0.054
Female		1,163 (58.6 %)	3367 (69.2 %)	241 (74.5 %)			
Race/ethnicity							
White non-Hispanic	At least 1 Year	367 (69.3 %)	2947 (78.4 %)	913 (78.5 %)	0.000	0.000	0.311
Black non-Hispanic		115 (8.9 %)	686 (7.6 %)	226 (8.5 %)			
Hispanic		211 (17.0 %)	803 (9.3 %)	258 (9.3 %)			
Other/multiple races		40 (4.8 %)	273 (4.8 %)	69 (3.7 %)			
White non-Hispanic	Both years	991 (71.6 %)	3028 (79.5 %)	208 (80.5 %)	0.000	0.017	0.584
Black non-Hispanic		296 (8.6 %)	690 (7.8 %)	41 (6.4 %)			
Hispanic		468 (14.5 %)	741 (8.4 %)	63 (9.7 %)			
Other/multiple races		118 (5.3 %)	254 (4.3 %)	10 (3.4 %)			
Marital status							
Married	At least 1 year	250 (31.6 %)	2100 (46.5 %)	793 (57.3 %)	0.000	0.000	0.000
Widowed		8 (1.6 %)	185 (3.5 %)	34 (2.1 %)			
Divorced/separated		144 (20.5 %)	1235 (25.0 %)	304 (19.4 %)			
Never married		331 (46.3 %)	1189 (25.0 %)	335 (21.2 %)			
Married	Both years	686 (35.9 %)	2266 (50.5 %)	191 (61.8 %)	0.000	0.000	0.012
Widowed		42 (2.2 %)	177 (3.4 %)	8 (1.9 %)			
Divorced/separated		419 (22.9 %)	1208 (24.0 %)	56 (17.2 %)			
Never married		726 (39.0 %)	1062 (22.2 %)	67 (19.0 %)			
Education							
Less than high school diploma/unknown	At least 1 year	204 (19.9 %)	935 (13.6 %)	226 (10.1 %)	0.000	0.000	0.027
High school diploma		236 (29.6 %)	1439 (28.9 %)	441 (28.9 %)			
Some college		172 (28.6 %)	1224 (28.5 %)	415 (31.0 %)			
4 years or more of college		121 (21.9 %)	1111 (29.0 %)	384 (30.0 %)			
Less than high school diploma/unknown	Both years	476 (18.3 %)	845 (12.1 %)	44 (7.6 %)	0.000	0.000	0.134
High school diploma		581 (28.6 %)	1445 (29.2 %)	90 (27.6 %)			
Some college		470 (29.5 %)	1247 (28.6 %)	94 (32.1 %)			
4 years or more of college		346 (23.6 %)	1176 (30.0 %)	94 (32.7 %)			
Family income - year 1							
Poor/near poor (less than 125 % of the federal poverty level (FPL))	At least 1 year	305 (34.6 %)	1413 (22.6 %)	320 (15.9 %)	0.000	0.000	0.000
Low income (125-200 % FPL)		155 (19.4 %)	701 (13.6 %)	219 (12.3 %)			
Middle income (200-400 % FPL)		179 (27.9 %)	1335 (29.6 %)	435 (30.9 %)			
High income (more than 400 % FPL)		94 (18.1 %)	1260 (34.2 %)	492 (40.9 %)			
Poor/near poor (less than 125 % FPL)	Both years	707 (29.6 %)	1277 (20.4 %)	54 (12.4 %)	0.000	0.000	0.029
Low income (125-200 % FPL)		357 (17.6 %)	669 (12.6 %)	49 (13.3 %)			
Middle income (200-400 % FPL)		489 (29.2 %)	1359 (29.8 %)	101 (30.9 %)			
High income (more than 400 % FPL)		320 (23.6 %)	1408 (37.3 %)	118 (43.4 %)			
Family income - year 2							
Poor/near poor (less than 125 % FPL)	At least 1 year	299 (34.1 %)	1433 (22.9 %)	332 (16.8 %)	0.000	0.000	0.001
Low income (125-200 % FPL)		114 (14.4 %)	679 (12.7 %)	216 (12.2 %)			
Middle income (200-400 % FPL)		214 (31.2 %)	1280 (27.8 %)	407 (28.6 %)			
High income (more than 400 % FPL)		106 (20.3 %)	1317 (36.6 %)	511 (42.4 %)			
Poor/near poor (less than 125 % FPL)	Both years	708 (30.3 %)	1290 (20.4 %)	66 (15.1 %)	0.000	0.000	0.149
Low income (125-200 % FPL)		322 (15.6 %)	651 (12.0 %)	36 (9.1 %)			
Middle income (200-400 % FPL)		517 (28.9 %)	1287 (27.9 %)	97 (31.9 %)			
High income (more than 400 % FPL)		326 (25.2 %)	1485 (39.8 %)	123 (44.0 %)			
Employment							
Never employed	At least 1 year	154 (17.4 %)	1679 (30.5 %)	404 (23.0 %)	0.000	0.000	0.000
Sometimes employed		269 (37.5 %)	1001 (20.1 %)	320 (22.3 %)			
Always employed		310 (45.1 %)	2029 (49.3 %)	742 (54.7 %)			
Never employed	Both years	468 (21.4 %)	1691 (30.3 %)	78 (21.9 %)	0.000	0.000	0.005
Sometimes employed		630 (32.5 %)	898 (18.9 %)	62 (17.4 %)			
Always employed		775 (46.2 %)	2124 (50.8 %)	182 (60.6 %)			
Health insurance status – year 1							
Any private insurance	At least 1 year	267 (45.9 %)	2672 (65.8 %)	967 (73.2 %)	0.000	0.000	0.000
Medicare		24 (3.0 %)	578 (10.9 %)	137 (8.2 %)			
Medicaid/other public coverage		110 (10.8 %)	830 (12.4 %)	217 (10.3 %)			
Uninsured		332 (40.4 %)	629 (10.9 %)	145 (8.4 %)			
Any private insurance	Both years	838 (54.6 %)	2836 (68.4 %)	232 (78.5 %)	0.000	0.000	0.004
Medicare		99 (5.1 %)	620 (11.5 %)	20 (5.6 %)			
Medicaid/other public coverage		318 (12.3 %)	790 (11.7 %)	49 (10.1 %)			
Uninsured		618 (28.0 %)	467 (8.4 %)	21 (5.8 %)			
Health insurance status – year 2							
Any private insurance	At least 1 year	259 (44.1 %)	2550 (63.2 %)	936 (71.0 %)	0.000	0.000	0.000
Medicare		30 (4.2 %)	672 (12.9 %)	154 (9.2 %)			
Medicaid/other public coverage		121 (12.1 %)	873 (13.1 %)	229 (11.4 %)			
Uninsured		323 (39.6 %)	614 (10.8 %)	147 (8.4 %)			
Any private insurance	Both years	790 (51.8 %)	2725 (66.0 %)	230 (78.6 %)	0.000	0.000	0.000
Medicare		115 (6.0 %)	717 (13.5 %)	24 (6.4 %)			
Medicaid/other public coverage		362 (14.3 %)	815 (12.3 %)	46 (8.9 %)			
Uninsured		606 (27.9 %)	456 (8.1 %)	22 (6.2 %)			
Mental health condition							
Anxiety disorders	At least 1 year	392 (54.0 %)	2595 (55.7 %)	806 (55.8 %)	0.447	0.484	0.936
Mood disorders		395 (53.9 %)	2955 (62.0 %)	872 (59.0 %)	0.001	0.066	0.094
Other mental health conditions		60 (8.7 %)	410 (8.3 %)	114 (8.2 %)	0.724	0.724	0.932
Anxiety disorders	Both years	1021 (53.9 %)	2594 (56.2 %)	178 (55.0 %)	0.135	0.775	0.744
Mood disorders		1081 (57.3 %)	2947 (61.7 %)	194 (60.2 %)	0.009	0.458	0.694
Other mental health conditions		166 (9.5 %)	401 (8.0 %)	17 (6.7 %)	0.098	0.198	0.498
Substance use disorder diagnosis							
Yes	At least 1 year	14 (1.9 %)	94 (2.3 %)	28 (1.8 %)	0.534	0.894	0.292
No		719 (98.1 %)	4,615 (97.7 %)	1,438 (98.2 %)			
Yes	Both years	42 (2.1 %)	88 (2.2 %)	6 (1.9 %)	0.722	0.879	0.734
No		1,831 (97.9 %)	4,625 (97.8 %)	316 (98.1 %)			

### Preventive care and healthcare quality

Two-way tables showed that provider type was associated with preventive care and healthcare quality in most analyses comparing participants who did not have a USC to either those who had a non-PCMH USC or those who received care consistent with the PCMH (Table 5). In contrast, most comparisons between participants who had a non-PCMH USC and those who received care consistent with the PCMH were not statistically significant. Specifically, comparisons between participants who did not have a USC and those who had a non-PCMH USC were statistically significant for all measures except foot exam in both years, eye exam in at least 1 year and both years, and follow-up after hospitalization for mental illness. Comparisons between participants who did not have a USC and those who received care consistent with the PCMH were statistically significant for all measures except eye exam in at least 1 year and both years and follow-up after hospitalization for mental illness. Comparisons between participants who had a non-PCMH USC and those who received care consistent with the PCMH were statistically significant for healthcare rating in at least 1 year and both years, cervical cancer screening in at least 1 year, current smoking in at least 1 year, and flu shot in at least 1 year. In nearly all instances in which there were statistically significant differences, the percentage of participants who met the preventive care or healthcare quality measure was higher for participants who had a non-PCMH USC compared to those who did not have a USC, as well as for participants who received care consistent with the PCMH compared to those who either had a non-PCMH USC or did not have a USC. The only exception to this finding was the current smoking measure, which showed the inverse relationship in all instances in which there were statistically significant relationships.

**Table 5:** Receipt of Preventive Care and Healthcare Quality by Provider Type

Receipt of Preventive Care/Healthcare Quality Measure			Provider Type			*P*-value		
			No USC N (weighted %)	Non-PCMH USC N (weighted %)	PCMH N (weighted %)	No USC vs. Non-PCMH USC	No USC vs. PCMH	Non-PCMH USC vs. PCMH
Healthcare rating (*N* = 4773)	At least 1 year	Yes	100 (46.4 %)	1986 (56.6 %)	728 (66.5 %)	0.018	0.000	0.000
		No	108 (53.6 %)	1489 (43.4 %)	362 (33.5 %)			
	Both years	Yes	133 (16.1 %)	1102 (28.9 %)	111 (45.4 %)	0.000	0.000	0.000
		No	696 (83.9 %)	2588 (71.1 %)	143 (54.6 %)			
Cervical cancer screening (*N* = 3133)	At least 1 year	Yes	234 (83.7 %)	1991 (92.3 %)	689 (95.9 %)	0.000	0.000	0.012
		No	44 (16.3 %)	148 (7.7 %)	27 (4.1 %)			
	Both years	Yes	614 (78.2 %)	1911 (87.4 %)	148 (90.0 %)	0.000	0.003	0.376
		No	177 (21.8 %)	266 (12.6 %)	17 (10.0 %)			
Breast cancer screening (*N* = 1461)	At least 1 year	Yes	31 (46.9 %)	916 (84.3 %)	275 (88.7 %)	0.000	0.000	0.092
		No	27 (53.1 %)	179 (15.7 %)	33 (11.3 %)			
	Both years	Yes	96 (50.3 %)	887 (74.9 %)	52 (75.9 %)	0.000	0.002	0.877
		No	97 (49.7 %)	313 (25.1 %)	16 (24.1 %)			
Colorectal cancer screening (*N* = 1531)	At least 1 year	Yes	24 (38.4 %)	805 (73.5 %)	266 (78.9 %)	0.000	0.000	0.078
		No	40 (61.6 %)	311 (26.5 %)	85 (21.1 %)			
	Both years	Yes	73 (36.2 %)	739 (63.5 %)	55 (66.3 %)	0.000	0.000	0.644
		No	145 (63.8 %)	487 (36.5 %)	32 (33.7 %)			
Current smoking (*N* = 6697)	At least 1 year	Yes	304 (45.8 %)	1497 (31.7 %)	412 (27.9 %)	0.000	0.000	0.036
		No	405 (54.2 %)	3065 (68.3 %)	1014 (72.1 %)			
	Both years	Yes	582 (32.9 %)	1097 (23.4 %)	68 (21.2 %)	0.000	0.000	0.455
		No	1234 (67.1 %)	3466 (76.6 %)	250 (78.8 %)			
Smoking cessation advice (*N* = 1382)	At least 1 year	Yes	83 (63.6 %)	861 (87.0 %)	230 (88.2 %)	0.000	0.000	0.623
		No	49 (36.4 %)	126 (13.0 %)	33 (11.8 %)			
	Both years	Yes	175 (46.6 %)	635 (67.0 %)	45 (76.0 %)	0.000	0.000	0.183
		No	204 (53.4 %)	307 (33.0 %)	16 (24.0 %)			
Flu shot (*N* = 6758)	At least 1 year	Yes	182 (25.2 %)	2469 (53.0 %)	816 (58.4 %)	0.000	0.000	0.004
		No	526 (74.8 %)	2146 (47.0 %)	619 (41.6 %)			
	Both years	Yes	283 (15.7 %)	1636 (36.1 %)	121 (40.2 %)	0.000	0.000	0.251
		No	1530 (84.3 %)	2994 (63.9 %)	194 (59.8 %)			
Foot exam (*N* = 640)	At least 1 year	Yes	9 (52.6 %)	401 (83.1 %)	111 (82.9 %)	0.006	0.013	0.946
		No	10 (47.4 %)	86 (16.9 %)	23 (17.1 %)			
	Both years	Yes	35 (48.4 %)	288 (53.7 %)	20 (75.9 %)	0.493	0.046	0.061
		No	45 (51.6 %)	244 (46.3 %)	8 (24.1 %)			
Eye exam (*N* = 643)	At least 1 year	Yes	13 (82.3 %)	421 (84.9 %)	117 (87.4 %)	0.729	0.536	0.561
		No	6 (17.7 %)	68 (15.1 %)	18 (12.6 %)			
	Both years	Yes	39 (53.2 %)	333 (64.9 %)	19 (66.0 %)	0.156	0.333	0.913
		No	41 (46.8 %)	201 (35.1 %)	10 (34.0 %)			
Follow-up after hospitalization for mental illness (*N* = 245)	At least 1 year	Yes	4 (12.3 %)	36 (30.4 %)	12 (16.3 %)	0.068	0.676	0.074
		No	28 (87.7 %)	120 (69.6 %)	45 (83.7 %)			

In multivariate models, after adjusting for multiple comparisons, participants who had a non-PCMH USC had significantly higher odds of meeting the following preventive care and healthcare quality measures compared to participants who did not have a USC: (1) healthcare rating in both years (AOR 1.96; 95 % CI 1.52, 2.53); (2) cervical cancer screening in at least 1 year (AOR 2.33; 95 % CI 1.41, 3.87) and both years (AOR 1.96; 95 % CI 1.46, 2.63); (3) breast cancer screening in both years (AOR 2.19; 95 % CI 1.45, 3.30); (4) smoking cessation advice in at least 1 year (AOR 2.87; 95 % CI 1.75, 4.70) and both years (AOR 1.81; 95 % CI 1.30, 2.52); and (5) flu shot in at least 1 year (AOR 1.88; 95 % CI 1.46, 2.43) and both years (AOR 1.83; 95 % CI 1.54, 2.18) (Table 6). Participants who had a non-PCMH USC also had significantly lower odds of current smoking in at least 1 year (AOR 0.66; 95 % 0.53, 0.82) compared to participants who did not have a USC.

**Table 6:** Odds of Preventive Care and Healthcare Quality by Provider Type

Preventive Care/Healthcare Quality Measure	At Least 1 Year		Both Years	
	Unadjusted OR OR (95 % CI)	Adjusted OR (95 % CI)	Unadjusted OR (95 % CI)	Adjusted OR (95 % CI)
Participants with a Non-PCMH USC Compared to Participants without a USC				
Healthcare rating	1.50 (1.07, 2.11)*	1.29 (0.90, 1.84)	2.13 (1.67, 2.71)***	1.96 (1.52, 2.53)****
Cervical cancer screening	2.33 (1.50, 3.64)***	2.33 (1.41, 3.87)****	1.92 (1.48, 2.51)***	1.96 (1.46, 2.63)****
Breast cancer screening	-	-	2.95 (1.99, 4.36)***	2.19 (1.45, 3.30)****
Current smoking	0.55 (0.46, 0.66)***	0.66 (0.53, 0.82)****	0.62 (0.53, 0.73)***	0.77 (0.64, 0.93)
Smoking cessation advice	3.83 (2.42, 6.06)***	2.87 (1.75, 4.70)****	2.33 (1.71, 3.19)***	1.81 (1.30, 2.52)****
Flu shot	3.35 (2.70, 4.15)***	1.88 (1.46, 2.43)****	3.04 (2.58, 3.59)***	1.83 (1.54, 2.18)****
Participants who Received Care Consistent with the PCMH Compared to Participants without a USC				
Healthcare rating	2.29 (1.57, 3.35)***	2.29 (1.53, 3.41)****	4.35 (2.93, 6.45)***	4.39 (2.82, 6.84)****
Cervical cancer screening	-	-	2.49 (1.35, 4.58)**	2.35 (1.23, 4.46)
Current smoking	0.46 (0.37, 0.57)***	0.73 (0.54, 0.99)	0.55 (0.40, 0.76)***	0.86 (0.57, 1.29)
Flu shot	4.16 (3.23, 5.36)***	3.00 (2.24, 4.04)****	3.63 (2.63, 5.00)***	2.28 (1.57, 3.31)****
Participants who Received Care Consistent with the PCMH Compared to Participants with a Non-PCMH USC				
Healthcare rating	1.53 (1.27, 1.84)***	1.46 (1.20, 1.79)****	2.04 (1.52, 2.74)***	2.07 (1.50, 2.86)****
Cervical cancer screening	1.95 (1.15, 3.31)*	1.65 (0.96, 2.83)	1.29 (0.73, 2.30)	1.10 (0.61, 1.99)
Breast cancer screening	1.46 (0.94, 2.29)	1.23 (0.75, 2.00)	1.06 (0.53, 2.10)	0.82 (0.38, 1.75)
Colorectal cancer screening	1.35 (0.97, 1.87)	1.24 (0.88, 1.75)	1.13 (0.67, 1.90)	1.08 (0.61, 1.91)
Current smoking	0.83 (0.70, 0.99)*	0.98 (0.81, 1.20)	0.88 (0.63, 1.23)	1.07 (0.74, 1.54)
Smoking cessation advice	1.12 (0.71, 1.75)	1.15 (0.74, 1.78)	1.56 (0.81, 3.02)	1.61 (0.84, 3.10)
Flu shot	1.24 (1.07, 1.44)**	1.22 (1.04, 1.43)	1.19 (0.88, 1.61)	1.27 (0.91, 1.77)
Participants who Received Care Consistent with the PCMH Compared to Participants who Did Not Receive Care Consistent with the PCMH^a^				
Cervical cancer screening	2.19 (1.29, 3.71)**	1.78 (1.04, 3.05)	-	-
Breast cancer screening	1.67 (1.07, 2.59)*	1.36 (0.85, 2.18)	1.25 (0.63, 2.47)	0.90 (0.40, 2.00)
Colorectal cancer screening	1.47 (1.06, 2.04)*	1.28 (0.90, 1.81)	1.34 (0.81, 2.24)	1.31 (0.75, 2.28)
Smoking cessation advice	1.37 (0.89, 2.11)	1.42 (0.94, 2.16)	1.99 (1.04, 3.79)*	1.87 (0.98, 3.57)
Foot exam	1.05 (0.62, 1.77)	1.03 (0.60, 1.76)	-	-
Eye exam	1.23 (0.62, 2.47)	1.06 (0.52, 2.16)	-	-
Follow-up after hospitalization for mental illness	0.50 (0.20, 1.24)	0.47 (0.20, 1.10)	-	-

*p < 0.05; **p < 0.01; ***p < 0.001

*P*-value corrected for multiple comparisons; corrected *p*-value = 0.0018; ***p < 0.0018

^a^ Participants who did not receive care consistent with the PCMH included participants with a non-PCMH USC and participants without a USC

Participants who received care consistent with the PCMH had significantly higher odds of meeting the following preventive care and healthcare quality measures compared to participants who did not have a USC: (1) healthcare rating in at least 1 year (AOR 2.29; 95 % CI 1.53, 3.41) and both years (AOR 4.39; 95 % CI 2.82, 6.84); and (2) flu shot in at least 1 year (AOR 3.00; 95 % CI 2.24, 4.04) and both years (AOR 2.28; 95 % CI 1.57, 3.31). There was also a trend towards higher odds of receiving cervical cancer screening in both years (AOR 2.35; 95 % CI 1.23, 4.46) for participants who received care consistent with the PCMH compared to participants who did not have a USC.

Participants who received care consistent with the PCMH had significantly higher odds of meeting the healthcare rating measure in at least 1 year (AOR 1.46; 95 % CI 1.20, 1.79) and both years (AOR 2.07; 95 % CI 1.50, 2.86) compared to participants who had a non-PCMH USC.

Differences between participants who received care consistent with the PCMH and participants who did not receive care consistent with the PCMH were not significant for any measures. In these analyses, participants who did not receive care consistent with the PCMH included participants with a non-PCMH USC and participants without a USC.

Sensitivity analyses that excluded participants who did not have the same provider type in both years produced comparable findings and did not result in changes to statistical significance in any models (Additional file 1: Table S1). However, some sensitivity analyses could not be conducted because of small sample sizes. Specifically, sensitivity analyses could not be conducted for the following measures: breast cancer screening and smoking cessation advice in the analyses comparing participants who had a non-PCMH USC to those who did not have a USC (two of six measures); healthcare rating, cervical cancer screening, and flu shot in the analyses comparing participants who received care consistent with the PCMH to those who did not have a USC (three of four measures); and no measures comparing participants who received care consistent with the PCMH to those with a non-PCMH USC.

## Discussion

As the first national study to assess the association between receipt of care consistent with the PCMH and preventive care and healthcare quality measures for non-elderly adults with mental illness, this study addresses an important gap in the literature. This study provides evidence that non-elderly adults with mental illness who have a non-PCMH USC or who receive care consistent with the PCMH may be more likely to receive recommended preventive care or better healthcare quality, on most measures, compared to non-elderly adults without a USC. However, it does not provide evidence that, compared to having a USC that does not meet PCMH criteria, receiving care consistent with the PCMH is associated with receipt of recommended preventive care or better healthcare quality for most measures.

More specifically, this study found that, compared to participants who did not have a USC, participants who had a non-PCMH USC had significantly better odds of receiving recommended preventive care and healthcare quality for almost all measures examined. Similarly, compared to participants who did not have a USC, participants who received care consistent with the PCMH had significantly better odds of receiving recommended preventive care and healthcare quality for most measures examined. In contrast, participants who received care consistent with the PCMH had significantly better odds of meeting only one preventive care or healthcare quality measure (i.e., healthcare rating) compared to participants with a non-PCMH USC. Differences between participants who received care consistent with the PCMH and participants who did not receive care consistent with the PCMH, which included participants with a non-PCMH USC and participants without a USC, were not significant for any measures examined. These findings indicate that non-elderly adults with mental illness who receive care consistent with the PCMH may rate their healthcare more favorably than non-elderly adults with mental illness who have a non-PCMH USC. However, receipt of care consistent with the PCMH does not appear to provide an incremental benefit over having a non-PCMH USC for most preventive care or healthcare quality measures for this population. This raises concerns about the potential value of the PCMH for non-elderly adults with mental illness and suggests that alternative models of care are needed to improve their health outcomes.

The results of this study contrast with those of prior studies which indicated that the PCMH may be associated with better medication adherence [19, 20], receipt of preventive screenings [20], and outpatient follow-up after psychiatric discharge [21] for non-elderly adults with mental illness. The differences in study findings, however, could be attributed to methodological differences between the studies.

First, this study used self-reported data to assess receipt of preventive care and healthcare quality measures, while the prior studies used claims and other encounter data [19–21]. There can be low rates of concordance between claims data and self-reported data [38], as well as potential problems with the accuracy and completeness of both types of data [38–40] for assessing healthcare quality. These issues could have contributed to differences in the results. Second, prior studies included participants who were enrolled in the North Carolina Medicaid program [19–21], while this study used a nationally representative sample of MEPS participants. Third, prior studies included participants with specific types of mental health conditions [19–21], focused on participants with multiple chronic conditions [19, 21], and/or stratified results by condition [20]. In comparison, this study included participants with all types of mental illness, did not stratify results by condition, and did not limit participants to people with multiple chronic conditions. Fourth, two of the prior studies [19, 20] examined medication adherence and follow-up after psychiatric hospitalization, which could not be examined in this study because of data limitations in MEPS and/or insufficient power in this study. Future studies should further examine whether the association between the PCMH and preventive care and/or healthcare quality varies by mental illness type and should assess preventive care and healthcare quality measures that could not be examined in this study.

While this study largely does not provide evidence to indicate that the PCMH offers preventive care or healthcare quality benefits over having a non-PCMH USC, sample sizes limited the number and breadth of preventive care and healthcare quality measures that could have been examined. Small sample sizes may also have resulted in large standard errors that prevented results for some measures from reaching statistical significance. Further, the lack of evidence to support an association between the PCMH overall and preventive care and healthcare quality measures does not necessarily indicate that individual PCMH attributes are not associated with better quality of care or that the PCMH is not associated with other types of healthcare measures. Additional studies should be conducted to assess the impact of individual PCMH attributes and to examine the relationship between having a PCMH and healthcare utilization, healthcare costs, and other preventive care and healthcare quality measures.

Some additional study limitations should be noted. First, due to limited data in MEPS, some PCMH attributes could not be assessed, and there may be some aspects of PCMH attributes that were not included in the PCMH categorization. Second, because our study did not require a PCMH to be a primary care provider, it was possible for a participant whose USC was a specialist to be classified as receiving care consistent with the PCMH. This could have impacted the results, as a mental health clinic that meets the PCMH criteria of this study may, for instance, be less focused on ensuring that patients receive recommended cancer screenings than a PCMH with a primary care specialty would be. Third, subjectivity in participant responses could have led care to be inappropriately classified as meeting or not meeting PCMH criteria. Further, some participants had one or more PCMH variables recoded to indicate that they did not receive the PCMH characteristic in each year because they did not know whether the USC met a characteristic of comprehensive, patient-centered, and/or accessible care. In recoding these participants, we assumed that a person should know that the USC met each characteristic if the USC was a PCMH. While we were careful to select PCMH variables that participants would be able to provide information on if the USC met the characteristic, it is possible for a participant to respond that he or she did not know the answer to a question because of recall issues. As a result, the recoding of the results could have led some participants to be inappropriately classified as not receiving care consistent with the PCMH. This could have biased the results towards the null. Additional studies are needed to validate a PCMH definition with MEPS data for research purposes.

Fourth, participants were identified as having mental illness if they self-reported currently experiencing a mental health condition and/or having a mental health condition linked to an event or disability day during the survey period. As a result, some participants could have been misclassified as not having mental illness. Fifth, missing data for some covariates were classified unknown and included in the analyses. This could have introduced some bias in the results. Sixth, this study was limited to non-elderly adults. There are substantial differences between younger and older adults (e.g., prevalence of mental illness [1], severity and types of mental health conditions [1], use of healthcare services [1, 41], social and economic factors [42, 43]) that warrant examining this topic separately for non-elderly and elderly adults. Additional studies focused on elderly adults are needed. Other study limitations are the observational design, use of secondary data, and use of proxy respondents.

## Conclusion

As the first national study to assess the association between receipt of care consistent with the PCMH and preventive care and healthcare quality measures for non-elderly adults with mental illness, this study addresses an important gap. This study provides evidence that non-elderly adults with mental illness who have a non-PCMH USC or who receive care consistent with the PCMH may be more likely to receive recommended preventive care or better healthcare quality on most measures, compared to non-elderly adults without a USC. However, it does not provide evidence that, compared to having a USC that does not meet PCMH criteria, receipt of care consistent with the PCMH is associated with receipt of recommended preventive care or better healthcare quality for most measures. Additional research is needed to better understand explanatory factors in the relationship between receipt of care consistent with the PCMH and preventive care and healthcare quality. Future research should explore the extent to which study findings may change based on study assumptions and methodology. Additional research is also needed to assess whether the association between the PCMH and preventive care and/or healthcare quality varies by mental illness type, to examine the association between the PCMH and additional preventive care and healthcare quality measures, to evaluate the impact of the PCMH on healthcare services utilization and costs, and to assess the impact of the PCMH for elderly adults.

## Additional file

Additional file 1: Table S1. Results of Sensitivity Analyses. The table presents the results of sensitivity analyses that excluded participants who did not have the same provider type in both years. These additional analyses focused on measures and time periods that could not be assessed in one or more of the main multivariate analyses because of small sample sizes. (DOCX 87 kb)

## Abbreviations

AHRQ: Agency for Healthcare Research and Quality
AOR: Adjusted odds ratio
CI: Confidence interval
ER: Emergency room
FPL: Federal poverty level
MEPS: Medical Expenditure Panel Survey
PCMH: Patient-centered medical home
SAMHSA: Substance Abuse and Mental Health Services Administration
US: United States
USC: Usual source of care
