# Supplementary material for: Associations between the patient-centered medical home and preventive care and healthcare quality for non-elderly adults with mental illness: A surveillance study analysis
Source: BMC Health Serv Res. 2016 Aug 24;16(1):434. doi: 10.1186/s12913-016-1676-z (PMC4997759; doi:10.1186/s12913-016-1676-z)
Supplement: Additional file 1: Table S1. — Results of Sensitivity Analyses. The table presents the results of sensitivity analyses that excluded participants who did not have the same provider type in both years. These additional analyses focused on measures and time periods that could not be assessed in one or more of the main multivariate analyses because of small sample sizes. (DOCX 87 kb) [file 12913_2016_1676_MOESM1_ESM.docx]

| **Additional file 1: Table S1. Results of Sensitivity Analyses** | | |
| --- | --- | --- |
| **Preventive Care/**  **Healthcare Quality Measure** | **Original Analyses** | **Sensitivity Analyses** |
|  | **Adjusted OR (95% CI)** | **Adjusted OR (95% CI)** |
| **Participants with a Non-PCMH USC Compared to Participants without a USC** | | |
| **Healthcare rating** | 1.96 (1.52, 2.53)*** | 1.92 (1.50, 2.47)*** |
| **Cervical cancer screening** | 1.96 (1.46, 2.63)*** | 2.07 (1.43, 3.01)*** |
| **Current smoking** | 0.77 (0.64, 0.93)** | 0.70 (0.54, 0.90)** |
| **Flu shot** | 1.83 (1.54, 2.18)*** | 1.98 (1.35, 2.90)*** |
| **Participants who Received Care Consistent with the PCMH Compared to Participants without a USC** | | |
| **Current smoking** | 0.86 (0.57, 1.29) | 0.95 (0.58, 1.55) |
| **Participants who Received Care Consistent with the PCMH Compared to**  **Participants with a Non-PCMH USC** | | |
| **Healthcare rating** | 2.07 (1.50, 2.86)*** | 2.13 (1.51, 3.00)*** |
| **Cervical cancer screening** | 1.10 (0.61, 1.99) | 1.17 (0.62, 2.15) |
| **Breast cancer screening** | 0.82 (0.38, 1.75) | 0.82 (0.38, 1.81) |
| **Colorectal cancer screening** | 1.08 (0.61, 1.91) | 1.02 (0.57, 1.83) |
| **Current smoking** | 1.07 (0.74, 1.54) | 1.04 (0.71, 1.52) |
| **Smoking cessation advice** | 1.61 (0.84, 3.10) | 1.55 (0.78, 3.06) |
| **Flu shot** | 1.27 (0.91, 1.77) | 1.32 (0.95, 1.84) |
| *p<0.05; **p<0.01; ***p<0.001 | | |
